# Rise of CC398 Lineage of *Staphylococcus aureus* among Infective Endocarditis Isolates Revealed by Two Consecutive Population-Based Studies in France

**DOI:** 10.1371/journal.pone.0051172

**Published:** 2012-12-13

**Authors:** Anne Tristan, Jean-Philippe Rasigade, Esmée Ruizendaal, Frédéric Laurent, Michèle Bes, Hélène Meugnier, Gérard Lina, Jerome Etienne, Marie Celard, Pierre Tattevin, Stefan Monecke, Vincent Le Moing, François Vandenesch, the French AEPEI study Group on Infective Endocarditis

**Affiliations:** 1 Hospices Civils de Lyon, Centre National de Référence des Staphylocoques, Bron, France; 2 Université de Lyon, Domaine de la Buire, Lyon, France; 3 INSERM U851, Bacterial Pathogenesis and Innate Immunity Laboratory, Lyon, France; 4 Infectious Diseases and Intensive Care Unit, Pontchaillou University Hospital, Rennes, France; 5 Institute for Medical Microbiology and Hygiene, Faculty of Medicine “Carl Gustav Carus”, Technical University of Dresden, Dresden, Germany; 6 Département des maladies infectieuses et tropicales, hôpital Gui-de-Chauliac, Montpellier, France; University of California, San Francisco, United States of America

## Abstract

*Staphylococcus aureus* isolates from two prospective studies on infective endocarditis (IE) conducted in 1999 and 2008 and isolated from non-IE bacteremia collected in 2006 were *spa*-typed and their virulence factors were analyzed with a microarray. Both populations were genetically diverse, with no virulence factors or genotypes significantly more associated with the IE isolates compared with the non-IE isolates. The population structure of the IE isolates did not change much between 1999 and 2008, with the exception of the appearance of CC398 methicillin-susceptible *Staphylococcus aureus* (MSSA) isolates responsible for 5.6% of all cases in 2008. In 1999, this lineage was responsible for no cases. The increasing prevalence of *S. aureus* in IE is apparently not the result of a major change in staphylococcal population structure over time, with the exception of the emerging CC398 MSSA lineage.

## Introduction

Infective endocarditis (IE) is a rare but severe disease, which characteristics have changed over the past decades, and a shift in causative microorganisms has been observed. Staphylococci have surpassed streptococci as the primary group of IE-causing pathogens [1], as confirmed by a number of observational studies [2], [3]. Our study group conducted three population-based studies of IE in 1991, 1999 and 2008, using the same methods, based on a quarter of the French population [4]–[6]. These studies revealed an increasing *S. aureus* prevalence over the two decades covered by those three individual year time points (16.1%, 21.1%, and 25.7% for 1991, 1999 and 2008, respectively) [4]. In the 2008 survey, although the streptococci as a group were more frequently observed, *S. aureus* was the leading single-species cause of IE. Moreover, healthcare-associated IE accounted for almost 25% of all IE cases [6]. This result is consistent with other studies that previously indicated a shift from IE, mostly of dental origin, to a predominance of healthcare-related infections [7]. These evolutions in the microbial epidemiology of IE have been attributed to population aging, changes in predisposing cardiac conditions, and bloodstream infection (BSI) patterns [8]. However, no study has examined whether a qualitative change of the microorganisms responsible for IE, such as a shift in the causative lineages or the emergence of new clones of *S. aureus*, has occurred over time.

Another important question concerns the specificity of infective endocarditis strains with respect to other *S. aureus* infections. When comparing methicillin-susceptible *S. aureus* (MSSA) from uncomplicated infections (uncomplicated bacteremia and soft tissue infections) with those isolated from IE or haematogenous bone and joint infections within the same hospital, Fowler *et al* found that CC5 and CC9 were associated with an increased severity of infection [9]. In another study comparing geographically matched complicated skin- and soft-tissue infection (SSTI) MSSA isolates with IE MSSA isolates, Nienaber *et al* found that CC30 was the sole genotype that was significantly more frequent in IE than in SSTI isolates [10]. However, the question remains as to whether *S. aureus* IE isolates differ from non-IE bacteremia isolates. Finally, one striking observation by Nienaber *et al* [10] is that the genes encoding toxic shock syndrome toxin-1 (TSST-1) and staphylococcal enterotoxin A (SEA), two major superantigens from *S. aureus*, were extremely prevalent in IE isolates from the United States (93.9% and 64.9% respectively). These results suggest that IE isolates harbor specific virulence factors that differ from those found in isolates sampled from people suffering other diseases.

In the present paper, we thoroughly analyzed a collection of *S. aureus* IE isolates collected in 2008 during a population-based survey that covered a quarter of French territory. To assess the temporal changes associated with IE-causing staphylococci, we compared the population structure of these isolates with those from a similar survey conducted in 1999. To determine the specificity of IE isolates versus non-IE bacteremia isolates we compared the population structure and the virulence gene content of IE isolates from the 2008 survey with those from non-IE bacteremia isolates collected throughout France in 2006–2007.

## Results

### Genotyping of IE MSSA isolates

The genotypes of the 89 MSSA IE isolates were analyzed both by *spa* typing and by the use of a microarray. The microarray assigned all isolates to clonal complexes (CCs). Overall, there was a large genotypic diversity as exemplified by the 18 different CCs detected. However, four CCs (CC45, CC5, CC15 and CC30) accounted for >50% of the 89 isolates (Table 1). Among the less frequent CCs, five isolates (5.6%) belonged to CC398. This assignment was verified by a CC398-specific PCR [11]. The genetic diversity of the IE isolates was further confirmed by *spa*-typing indicating that the 89 isolates were distributed among 63 *spa* types and 11 *spa* clusters, with 5 *spa* clusters accounting for >50% of the isolates.

**Table 1:** A comparison of the population structures of methicillin-susceptible *Staphylococcus aureus* isolates from patients with infective endocarditis (IE) or bloodstream infection (BSI) without IE^a^.

MLST Clonal complex (CC)^b^	IE isolates (%), n = 89	non-IE BSI isolates (%), n = 81	P-value^c^
CC45	16 (18.0)	14 (17.3)	1.000
CC5	16 (18.0)	15 (18.5)	1.000
CC15	11 (12.4)	4 (4.9)	0.108
CC30	11 (12.4)	11 (13.6)	0.823
CC8	8 (9.0)	10 (12.3)	0.619
CC398	5 (5.6)	2 (2.5)	0.447
Others	22 (24.7)	25 (30.9)	0.395

a IE and non-IE isolates were collected in 2008 and 2006, respectively.

b MLST Clonal Complexes were inferred from microarray analysis.

c P-values were calculated for each CC using a two-tailed Fisher's exact test. The P-value for the whole contingency table was 0.592.

**Figure 1:**
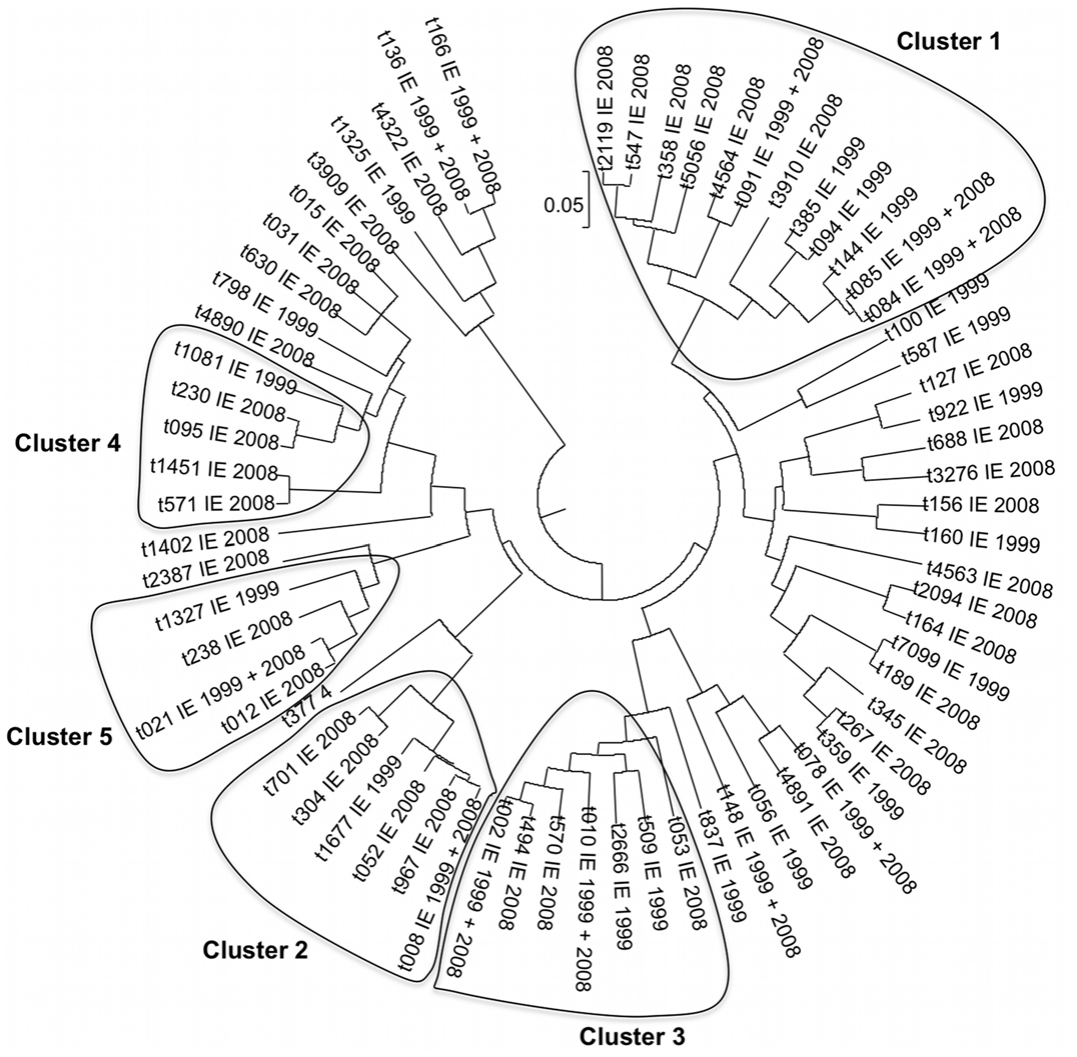
Phylogenetic tree based on *spa* type of *S. aureus* IE isolates from 1999 and 2008 surveys. Figure 1. Circular representation of the phylogenetic tree obtained using UPGMA method (http://spa.ridom.de/). The *spa* types were clustered into CCs (ie, *spa*CCs) by use of the integrated BURP (Based Upon Repeat Patterns) algorithm. User-definable parameters were set as follows: “cluster *spa* types into *spa*CC if cost distances are less than or equal to 4” and “exclude *spa* types shorter than 5 repeats.” This parameter combination ensures optimal concordance (95.3%) between BURP and e- BURST (http://spa.ridom.de/). Only major *spa*CC are represented.

To determine whether the population structure of the IE isolates was stable over time, the *spa* types of the *S. aureus* isolates collected during the 1999 French IE survey were determined. The results indicated that the two groups of isolates were genotypically very comparable with the *spa* types of the 2008 isolates interspaced with those of 1999 isolates (Figure 1). The exception was CC398, as none of the *spa* cluster identified in 1999 could be assigned to CC398.

### Correlation between CC and disease parameters

In order to determine whether certain lineages of *S. aureus* could be associated with certain clinical characteristics of the patients, search of associations between CC (assigned by the microarrays) and the patient database of the 2008 survey [6] was performed. The only significant association detected was between intravenous drug addiction and CC8. Four of the 8 CC8 isolates (50.0%) versus 14 of the 81 non-CC8 isolates (17.3%) were associated with IV drug abuse (*P*<0.05, two-tailed Fisher's exact test).

### Comparison of IE to non-IE blood-culture MSSA isolates

**Figure 2:**
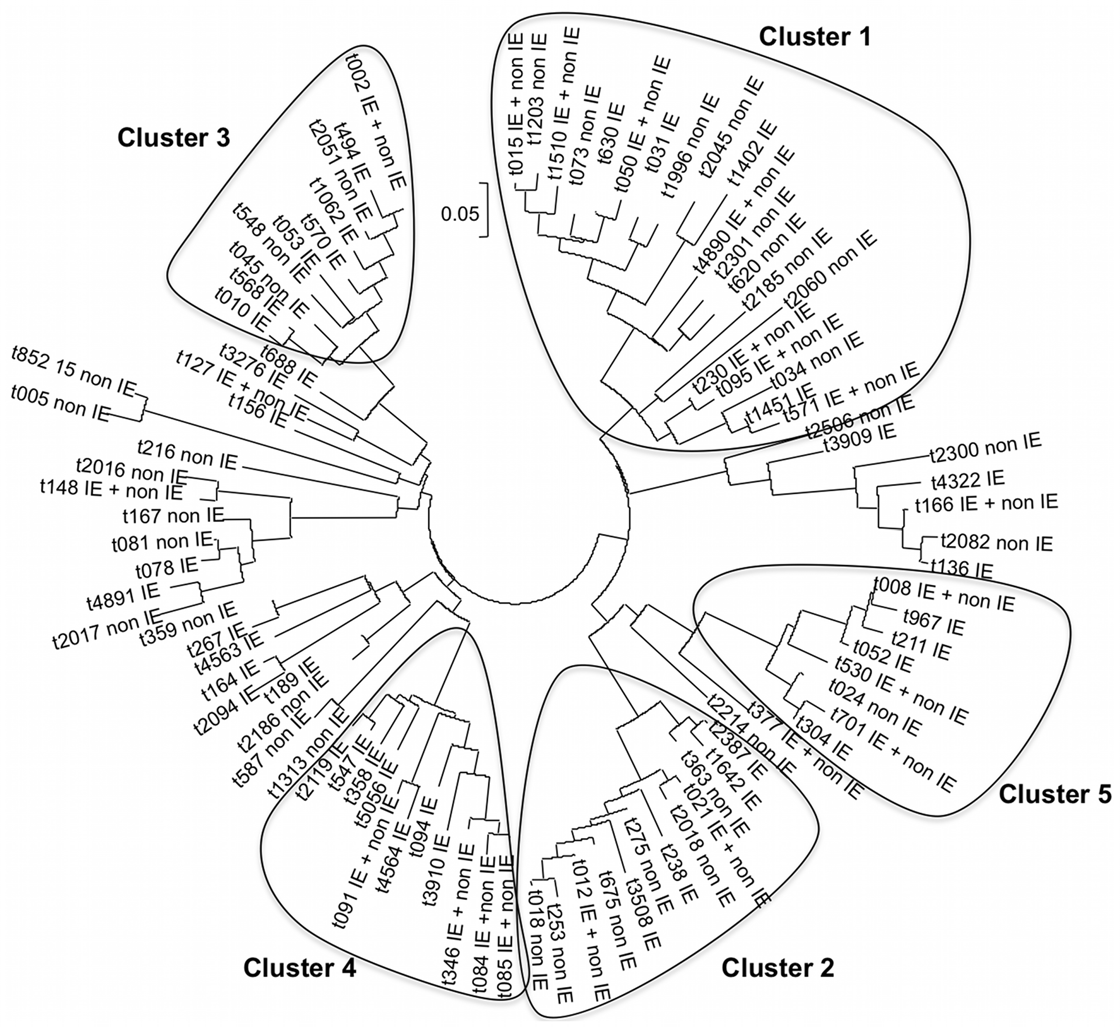
Phylogenetic tree based on *spa* type of *S. aureus* IE isolates (2008 survey) and bloodstream infections without IE (2006 survey). See Figure 1 legend for details.

To assess whether IE isolates correspond to peculiar lineages and harbor specific virulence factors in comparison to non-IE isolates, the population structure of the 89 MSSA IE isolates of the 2008 survey was compared with that of the 81 blood culture non-IE isolates collected in France during 2006. Genotypic comparison, as assessed both by *spa* typing and microarrays, revealed that the two populations were genotypically superimposable in regard to CCs (inferred from microarrays) (Table 1) and *spa* clusters (Figure 2). Figure 2 depicts that each *spa* cluster contained both IE and non-IE isolates. The two isolate populations were then compared to determine the prevalence of individual virulence factor genes. Genomic DNA microarray analysis comparison of the 89 MSSA IE isolates with the 81 non-IE bacteremia isolates indicated that there were no specific genes (including virulence factor genes) that could be significantly assigned to IE versus bacteremia isolates (Table 2 and Table S1). The known adhesin genes present in at least 60% of isolates of both groups were *fnb*AB (fibronectin binding protein A–B), *clf*AB (clumping factor A–B), *spa* (protein A), *sdr*CD (ser-asp rich fibrinogen-binding, bone sialoprotein-binding protein C and D), *bbp* (bone sialoprotein-binding protein), *ebp*S (cell wall associated fibronectin-binding protein) and *map* (major histocompatibility complex class II analog protein). Similarly, the exotoxins and other putative virulence factors present in at least 60% of isolates in both groups were *seg/sei* (encoding the *egc* cluster), *ica*A (intercellular adhesion protein) and *chp* (chemotaxis inhibitory protein) (Table 2).

**Table 2:** A comparison of the genotypic profiles of methicillin-susceptible *Staphylococcus aureus* isolates from patients with infective endocarditis (IE) or bloodstream infection (BSI) without IE^a^.

Gene or allele	IE isolates (%), n = 89	non-IE BSI isolates (%), n = 81	P-value^b^
**Adhesins**			
*fnbA*	89 (100.0)	79 (97.5)	0.226
*fnbB*	76 (85.4)	70 (86.4)	1.000
*clfA*	89 (100.0)	81 (100.0)	1.000
*clfB*	89 (100.0)	81 (100.0)	1.000
*cna*	37 (41.6)	33 (40.7)	1.000
*spa*	89 (100.0)	81 (100.0)	1.000
*sdrC*	89 (100.0)	81 (100.0)	1.000
*sdrD*	73 (82.0)	62 (76.5)	0.449
*bbp*	78 (87.6)	78 (96.3)	0.051
*ebpS*	89 (100.0)	81 (100.0)	1.000
*map/eap*	85 (95.5)	80 (98.8)	0.370
**Toxins**			
*eta*	0 (0.0)	1 (1.2)	0.476
*etb*	0 (0.0)	0 (0.0)	1.000
*tst*	8 (9.0)	16 (19.8)	0.050
*sea*	16 (18.0)	17 (21.0)	0.699
*seb*	1 (1.1)	5 (6.2)	0.104
*sec*	16 (18.0)	12 (14.8)	0.680
*sed*	5 (5.6)	4 (4.9)	1.000
*see*	0 (0.0)	0 (0.0)	1.000
*seg*	52 (58.4)	55 (67.9)	0.209
*seh*	6 (6.7)	6 (7.4)	1.000
*sei*	51 (57.3)	56 (69.1)	0.116
*sej*	5 (5.6)	4 (4.9)	1.000
*pvl*	0 (0.0)	0 (0.0)	1.000
*hla*	84 (94.4)	81 (100.0)	0.060
**Other putative virulence genes**			
*icaA*	89 (100.0)	81 (100.0)	1.000
*chp*	56 (62.9)	53 (65.4)	0.751
***agr*** ** alleles**			
*agr* I	44 (49.4)	42 (51.9)	0.761
*agr* II	30 (33.7)	22 (27.2)	0.406
*agr* III	14 (15.7)	17 (21.0)	0.429
*agr* IV	1 (1.1)	0 (0.0)	1.000

a IE and non-IE isolates were collected in 2008 and 2006, respectively.

b P-values were calculated for each gene or allele using a two-tailed Fisher's exact test.

## Discussion

A wide range of different *S. aureus* clonal complexes were found to be involved in IE. Altogether, 18 different CCs were detected. This distribution did not vary much over time as assessed by the *spa*-typing comparison of the 1999 and 2008 isolates (Figure 1). This observation suggests that the increased prevalence of *S. aureus* accompanying endocarditis is not due to a change in bacterial population structure but rather mainly due to non-bacterial factors, including population aging and an increase in conditions favoring *S. aureus* endocarditis, such as the use of prosthetic valves, pace-makers and hospitalization [6]. An exception to the general stability of the *S. aureus* population structure over time is the identification of five CC398 strains in the 2008 survey. CC398 includes MSSA and methicillin-resistant *S. aureus* (MRSA), the latter being associated with livestock-associated infections, mainly in pigs [12], although cases of infections in humans with this lineage have been described [13]. Only limited documentation of CC398 MSSA strains exists [14]. However, with a frequency of 5 out of 89 isolates (5.6%), CC398 MSSA cannot be neglected as an important IE pathogen in humans. Other reports suggest that CC398 is a new emerging pathogenic lineage in humans [13], [15]–[17]. Future studies are needed to determine whether this lineage will continue to expand throughout the population.

When comparing IE versus non-IE isolates from the same geographic area, we found no difference in the population structure between the IE and non-IE bacteremia isolates. CC5 and CC45 dominate in both populations and CC398 was equally prevalent in IE and non-IE isolates (Table 1). This result is consistent with other studies, which also found highly heterogenic strains in *S. aureus* infection without any particular CC dominating involvement in invasive infections [18]. Of note, CC30 was slightly less represented in the two populations (13.6% in IE, 12.4% in non-IE infection) than it was in the Nienaber study (19.5% in IE) [10].

The population structure of our IE and non-IE isolates does not appear to be different from that observed with other invasive or colonizing isolate series. For instance, the population structure of MSSA from 943 invasive infections in the Netherland over a period of 11 years revealed that a majority of isolates belonged to the same CCs as our series [18]. Interestingly, nasal carriage isolates from a collection of 155 carriers (152 MSSA and 3 MRSA) sampled in Germany belonged, in a decreasing order of prevalence, to CC8, CC30, CC15 and CC45, which correspond to four of the five most prevalent CCs in our series [19]. Likewise, when comparing CC affiliations of IE isolates to *S. aureus* isolate typing data obtained from healthy carriers or bone infections in Germany [19], [20], a similar pattern can be observed. In line with the lack of correlation between CC and diseases vs carriage, we found no major association between CC and clinical characteristics of the infective endocarditis with the exception of CC8 with IV drug abuse. Although significant, this association relies on a small population. In the context of IV drug abuse, this association may reflect a propensity of the CC8 lineage to better colonize the skin.

When considering the virulence factors potentially associated with IE, a number of staphylococcal adhesins are considered to play a role in the pathogenesis of infective endocarditis, both *in vitro* or when using animal experimental models. This list includes clumping factor A–B [21], [22], fibronectin-binding protein A–B [21], collagen-binding protein [23], SdrD/E [22] and polysaccharide intercellular adhesin [24]. In our series of IE isolates, these factors, with the exception of collagen-binding protein gene (*cna*), were highly prevalent; however, they were equally prevalent in non-IE bacteremia isolates (Table 2), suggesting that they might be required but are not sufficient for the development of IE. All other variable virulence genes were equally prevalent in the two populations (Table S1). Altogether, it appears that the population structure and prevalence of variable virulence genes are not significantly different between invasive isolates responsible for bloodstream infections. These observations do not rule out the possibility of differential expression of virulence factors between isolates as suggested by some studies [25].

As mentioned in the introduction, TSST-1 and SEA-encoding gene were extremely prevalent in IE isolates from the United States (93.9% and 64.9% respectively) [10]. These results are quite different from our epidemiology for both our IE isolates (9% *tst* and 18% *sea*) and non-IE bacteremia isolates (19.8% *tst* and 21% *sea*), as well as different from the collection of nasal carriage studied by Monecke *et al* with the same arrays (14.84% *tst* and 17.42% *sea*) [19]. As mentioned by Nienaber, the high prevalence of major superantigens in their series could “reflect linkage disequilibrium with unidentified virulence genes and represent a ‘biomarker’ of *S. aureus* isolates with an increased risk for IE rather than a causal association” [10]. Whole-genome sequencing of these isolates is currently under way (V. Fowler, personal communication) and may address this important issue. Finally, Panton-Valentine leukocidin (PVL) was not detected in any IE or bloodstream infection isolates from our series and was detected at a frequency below 20% in the US series [10].

There is one limitation to the present study. The strains collected from the non-IE patients were retrieved from a survey in which IE was not formerly ruled out by performing a trans-esophageal echocardiography (TEE) [26]. Thus, a few cases of IE may have been misdiagnosed in the present non-IE bacteremia series. However, given the expected prevalence of IE within the bacteremia isolates from various studies (5–10% in healthcare-related, 10 to 15% in community-acquired *S. aureus* bacteremia) [27], [28], this bias is unlikely to have had a significant impact on our observation that IE and non-IE isolates carry similar genetic backgrounds and virulence profile genes. However, a current prospective study matching IE and non-IE cases, both ascertained by the performance of a TEE, will provide a definite answer to this important question.

In conclusion, our data reveal that there was a wide genetic diversity within MSSA strains, with no virulence factors or CC particularly more involved in IE as compared to non-IE bacteremia, suggesting that the occurrence of infective endocarditis during *S. aureus* bacteremia depends essentially on host factors. The population structure of IE isolates changed little between the two surveys performed 9 years apart with the exception of an emerging phenomenon, the rise of the CC398 MSSA lineage as a cause of invasive infections.

## Materials and Methods

### Bacterial strains

#### IE Isolates

A collection of 89 MSSA, and 39 MSSA IE non-duplicate isolates collected during respective 2008 [6] and 1999 [5] population-based surveys was included in the study. These isolates were from confirmed cases of MSSA IE as determined by the modified Duke criteria [29]. Both one-year surveys (1999 and 2008) were conducted using the same methods. However, the 2008 study covered a larger area of French territory than the 1999 survey. Hence, when comparing the isolates of the two studies, the 2008 collection was restricted to the 78 MSSA isolates collected in the same area as the 1999 survey.

#### Non-IE isolates

Eighty-one blood culture isolates out of 116 invasive infection isolates (all MSSA) collected during a prospective multicenter study from September 2006 to February 2007 by 23 representative French hospital laboratories were included [26].

### Genotyping methods

Bacterial DNA was extracted according to the manufacturer's recommended protocol using commercial extraction kits (Qiagen). The diagnostic DNA microarrays, Identibac *S. aureus* Genotyping ® (Alere) used for this study, as well as related procedures and protocols, have been previously described in detail [19]. This microarray covers 332 different target sequences corresponding to approximately 185 distinct genes and their allelic variants. The assignation of isolates to clonal complexes (CCs) was determined by the comparison of hybridization profiles to previously typed multilocus sequence typing (MLST) reference strains [19].

A DNA sequence-based analysis of the protein A gene variable region was performed as previously described [30] using the nomenclature as described on the Ridom website (http://spa.ridom.de/). To infer clonal relatedness, the Based Upon Repeat Pattern (BURP) algorithm was used followed by circular representation of the phylogenetic tree obtained using UPGMA method (http://spa.ridom.de/) [31].

### Clinical characteristics of patient

The patient information of the 2008 survey had been collected prospectively by use of a standardized case report form as described [6]. The following variable were collected: sex, date of birth, living place, date of first symptoms and of first hospitalization, transfer from/to another facility, history of heart disease, comorbidities, Charlson index, procedures and situations at risk for IE, signs and symptoms of IE, echocardiographic data, microbiological data, laboratory and imaging investigations, medical and surgical treatment, and outcome [6].

### Statistical analysis

Differences in the distributions of genes, alleles, genotypes and clinical features were tested for significance using a two-tailed Fisher's exact test. Contingency tables larger than 2×2 used in population structure comparisons were first analyzed as a whole, then each proportion was individually tested for significance. To reflect the exploratory nature of the analysis, P-values were not corrected for multiple testing. P-values of <0.05 were considered to be statistically significant. Statistical analyses were performed using R software version 2.14.2 (The R Foundation for Statistical Computing, Vienna, Austria).

## Supporting Information

Table S1 **A comparison of the distributions of genes or alleles in methicillin-susceptible** ***Staphylococcus aureus isolates*** **from patients with infective endocarditis (IE) or bloodstream infection (BSI) without IE (only genes not shown in Table1 are presented).**
(DOCX)
