# Supplementary material for: Rise of CC398 Lineage of Staphylococcus aureus among Infective Endocarditis Isolates Revealed by Two Consecutive Population-Based Studies in France
Source: PLoS One. 2012 Dec 13;7(12):e51172. doi: 10.1371/journal.pone.0051172 (PMC3521771; doi:10.1371/journal.pone.0051172)
Supplement: Table S1 — A comparison of the distributions of genes or alleles in methicillin-susceptible Staphylococcus aureus isolates from patients with infective endocarditis (IE) or bloodstream infection (BSI) without IE (only genes not shown in Table1 are presented). (DOCX) [file pone.0051172.s001.docx]

| **Table S1.** A comparison of the distributions of genes or alleles in methicillin-susceptible *Staphylococcus aureus isolates* from patients with infective endocarditis (IE) or bloodstream infection (BSI) without IE^a^. | | | |
| --- | --- | --- | --- |
| Gene or allele^b^ | IE isolates (%), n=89 | Non-IE BSI isolates (%), n=81 | P-value^c^ |
| **SPECIES MARKER** |  |  |  |
| *rrn*D1 (domaine 1 of 23S-rRNA) | 89 (100.0) | 81 (100.0) | 1.000 |
| *gap*A | 88 (98.9) | 81 (100.0) | 1.000 |
| *kat*A | 87 (97.8) | 81 (100.0) | 0.498 |
| *Co*A | 88 (98.9) | 80 (98.8) | 1.000 |
| *nuc*1 | 87 (97.8) | 81 (100.0) | 0.498 |
| *sbi* | 86 (96.6) | 81 (100.0) | 0.247 |
| **REGULATORY GENES** |  |  |  |
| *sar*A | 89 (100.0) | 81 (100.0) | 1.000 |
| *sae*S | 89 (100.0) | 81 (100.0) | 1.000 |
| *vra*S | 89 (100.0) | 80 (98.8) | 0.476 |
| *hld* | 88 (98.9) | 81 (100.0) | 1.000 |
| **RESISTANCE : PENICILLINASE** |  |  |  |
| *bla*Z | 75 (84.3) | 58 (71.6) | 0.062 |
| **RESISTANCE : MLS-ANTIBIOTICS** |  |  |  |
| *erm*A | 1 (1.1) | 6 (7.4) | 0.055 |
| *erm*B | 0 (0.0) | 1 (1.2) | 0.476 |
| *erm*C | 4 (4.5) | 3 (3.7) | 1.000 |
| *lin*A | 0 (0.0) | 0 (0.0) | 1.000 |
| *msr*A | 3 (3.4) | 2 (2.5) | 1.000 |
| *mef*A | 0 (0.0) | 0 (0.0) | 1.000 |
| *mpb*BM | 1 (1.1) | 0 (0.0) | 1.000 |
| *vatA* | 1 (1.1) | 0 (0.0) | 1.000 |
| *vatB* | 0 (0.0) | 0 (0.0) | 1.000 |
| *vga* | 0 (0.0) | 1 (1.2) | 0.476 |
| *vga*A | 0 (0.0) | 0 (0.0) | 1.000 |
| *vgb* | 1 (1.1) | 0 (0.0) | 1.000 |
| **RESISTANCE : AMINOGLYSOSIDES** |  |  |  |
| *aac*A*-aph*D | 0 (0.0) | 0 (0.0) | 1.000 |
| *aad*D | 0 (0.0) | 0 (0.0) | 1.000 |
| *aph*A3 | 1 (1.1) | 0 (0.0) | 1.000 |
| **RESISTANCE : MISCELLANEOUS GENES** | |  |  |
| *sat* | 1 (1.1) | 0 (0.0) | 1.000 |
| *dfr*A | 0 (0.0) | 0 (0.0) | 1.000 |
| *far*1 | 1 (1.1) | 0 (0.0) | 1.000 |
| Q6GD50 (putat. fusidic acid resist.) | 3 (3.4) | 0 (0.0) | 0.247 |
| *mup*R | 0 (0.0) | 0 (0.0) | 1.000 |
| *tet*K | 3 (3.4) | 1 (1.2) | 0.622 |
| *tet*M | 0 (0.0) | 1 (1.2) | 0.476 |
| *cat* (total) | 1 (1.1) | 1 (1.2) | 1.000 |
| *cfr* | 0 (0.0) | 0 (0.0) | 1.000 |
| *fex*A | 0 (0.0) | 0 (0.0) | 1.000 |
| **RESISTANCE : EFFLUX SYSTEMS** |  |  |  |
| *qac*A | 3 (3.4) | 2 (2.5) | 1.000 |
| *qac*C (total) | 4 (4.5) | 5 (6.2) | 0.738 |
| **RESISTANCE : GLYCOPEPTIDES** |  |  |  |
| *van*A | 0 (0.0) | 0 (0.0) | 1.000 |
| *van*B | 0 (0.0) | 0 (0.0) | 1.000 |
| *van*Z | 0 (0.0) | 0 (0.0) | 1.000 |
| **VIRULENCE : ENTEROTOXINS** |  |  |  |
| *sek* | 2 (2.2) | 3 (3.7) | 0.670 |
| *sel* | 15 (16.9) | 12 (14.8) | 0.834 |
| *sem* | 50 (56.2) | 56 (69.1) | 0.113 |
| *sen* | 52 (58.4) | 56 (69.1) | 0.155 |
| *seo* | 49 (55.1) | 56 (69.1) | 0.082 |
| *seq* | 2 (2.2) | 3 (3.7) | 0.670 |
| *ser* | 1 (1.1) | 4 (4.9) | 0.193 |
| *seu* | 52 (58.4) | 54 (66.7) | 0.342 |
| ORF CM14 | 2 (2.2) | 0 (0.0) | 0.498 |
| **VIRULENCE : HLG AND LEUKOCIDINS** |  |  |  |
| *luk*F (*hlg*B) | 89 (100.0) | 81 (100.0) | 1.000 |
| *luk*S (*hlg*C) | 89 (100.0) | 81 (100.0) | 1.000 |
| *hlg*A | 86 (96.6) | 81 (100.0) | 0.247 |
| *luk*F-P83/*luk*M | 0 (0.0) | 4 (4.9) | 0.050 |
| *luk*D | 51 (57.3) | 41 (50.6) | 0.442 |
| *luk*E | 36 (40.4) | 40 (49.4) | 0.281 |
| **VIRULENCE : HLB-CONV PHAGES** |  |  |  |
| *sak* | 64 (71.9) | 68 (84.0) | 0.067 |
| *scn* | 78 (87.6) | 74 (91.4) | 0.465 |
| **VIRULENCE : EXFOL.TOXINS** |  |  |  |
| *etd* | 2 (2.2) | 5 (6.2) | 0.260 |
| **VIRULENCE : EPITHEL. DIFF. INHIB** |  |  |  |
| *edin*A | 0 (0.0) | 0 (0.0) | 1.000 |
| *edin*B | 2 (2.2) | 7 (8.6) | 0.088 |
| edinC | 0 (0.0) | 0 (0.0) | 1.000 |
| **VIRULENCE : ACME LOCUS** |  |  |  |
| ACME | 0 (0.0) | 0 (0.0) | 1.000 |
| **VIRULENCE : PROTEASES** |  |  |  |
| *aur* | 89 (100.0) | 80 (98.8) | 0.476 |
| *spl*A | 53 (59.6) | 41 (50.6) | 0.281 |
| *spl*B | 53 (59.6) | 41 (50.6) | 0.281 |
| *spl*E | 41 (46.1) | 42 (51.9) | 0.539 |
| **CAPSULE -ASSOCIATED GENES** | |  |  |
| capsule type 1 | 0 (0.0) | 0 (0.0) | 1.000 |
| capsule type 5 | 37 (41.6) | 41 (50.6) | 0.281 |
| capsule type 8 | 52 (58.4) | 40 (49.4) | 0.281 |
| **ADHESION FACTORS / MSCRAMM GENES** | |  |  |
| *ebh* (cons) | 89 (100.0) | 78 (96.3) | 0.106 |
| *eno* | 89 (100.0) | 81 (100.0) | 1.000 |
| *sas*G (total) | 46 (51.7) | 38 (46.9) | 0.543 |
| *vwb* (total) | 89 (100.0) | 81 (100.0) | 1.000 |
| ^a^IE and non-IE isolates were collected in 2008 and 2006, respectively.  ^b^only the genes not shown in Table 1 are presented excluding SCC*mec*-related genes. ^c^P-values were calculated for each gene or allele using a two-tailed Fisher's exact test. | | | |
